# Supplementary material for: Discovery and application of insertion-deletion (INDEL) polymorphisms for QTL mapping of early life-history traits in Atlantic salmon
Source: BMC Genomics. 2010 Mar 8;11:156. doi: 10.1186/1471-2164-11-156 (PMC2838853; doi:10.1186/1471-2164-11-156)
Supplement: Additional file 2 — Information on developed 76 locus single-run INDEL panel in Atlantic salmon. Information on fluorescence labeling, primer concentrations, PCR pooling and links to alignments, INDEL motifs and GENESCAN (Burge and Karlin 1997) predictions of genes/exons are available in html format. [file 1471-2164-11-156-S2.ZIP › Additionalfile2/snpsummary4955.html]

```
Cluster 1210 Contig 5

prev  Summary    Contig List  next
```

Size of Consensus sequence = 1069

Number of sequences = 15

Minimum redundancy = 5

Key

A gi|117473709|gb|EG805928.1|EG805928 EST\_ssal\_evd\_6655 ssalevd thymus Salmo salar cDNA Salmo salar cDNA clone ssal\_evd\_507\_231\_fwd 3', mRNA sequence  
B gi|117841009|gb|EG913705.1|EG913705 EST\_ssal\_evf\_15493 ssalevf mixed\_tissue Salmo salar cDNA Salmo salar cDNA clone ssal\_evf\_519\_092\_fwd 3', mRNA sequence  
C gi|117514004|gb|EG845763.1|EG845763 EST\_ssal\_eve\_58479 ssaleve thyroid Salmo salar cDNA Salmo salar cDNA clone ssal\_eve\_579\_270\_fwd 3', mRNA sequence  
D gi|117446336|gb|EG778557.1|EG778557 EST\_ssal\_evd\_2106 ssalevd thymus Salmo salar cDNA Salmo salar cDNA clone ssal\_evd\_501\_166\_fwd 3', mRNA sequence  
E gi|117520297|gb|EG852025.1|EG852025 EST\_ssal\_eve\_13015 ssaleve thyroid Salmo salar cDNA Salmo salar cDNA clone ssal\_eve\_517\_267\_rev 5', mRNA sequence  
F gi|117514003|gb|EG845762.1|EG845762 EST\_ssal\_eve\_58478 ssaleve thyroid Salmo salar cDNA Salmo salar cDNA clone ssal\_eve\_579\_270\_rev 5', mRNA sequence  
G gi|117473708|gb|EG805927.1|EG805927 EST\_ssal\_evd\_6654 ssalevd thymus Salmo salar cDNA Salmo salar cDNA clone ssal\_evd\_507\_231\_rev 5', mRNA sequence  
H gi|117446325|gb|EG778546.1|EG778546 EST\_ssal\_evd\_2105 ssalevd thymus Salmo salar cDNA Salmo salar cDNA clone ssal\_evd\_501\_166\_rev 5', mRNA sequence  
I gi|117849127|gb|EG921823.1|EG921823 EST\_ssal\_evf\_997 ssalevf mixed\_tissue Salmo salar cDNA Salmo salar cDNA clone ssal\_evf\_005\_170\_rev 5', mRNA sequence  
J gi|117841010|gb|EG913706.1|EG913706 EST\_ssal\_evf\_15494 ssalevf mixed\_tissue Salmo salar cDNA Salmo salar cDNA clone ssal\_evf\_519\_092\_rev 5', mRNA sequence  
K gi|117468343|gb|EG800562.1|EG800562 EST\_ssal\_evd\_57288 ssalevd thymus Salmo salar cDNA Salmo salar cDNA clone ssal\_evd\_577\_101\_rev 5', mRNA sequence  
L gi|84971540|gb|DW469941.1|DW469941 SGP298820 Atlantic salmon Testis cDNA library Salmo salar cDNA clone MG4-3157 5', mRNA sequence  
M gi|89855996|gb|DY712119.1|DY712119 EST\_ssal\_rgb2\_67858 ssalrgb2 mixed\_tissue Salmo salar cDNA Salmo salar cDNA clone ssal\_rgb2\_610\_103\_fwd 3', mRNA sequence  
N gi|117534050|gb|EG865495.1|EG865495 EST\_ssal\_eve\_27137 ssaleve thyroid Salmo salar cDNA Salmo salar cDNA clone ssal\_eve\_536\_344\_rev 5', mRNA sequence  
O gi|117479494|gb|EG811711.1|EG811711 EST\_ssal\_evd\_18225 ssalevd thymus Salmo salar cDNA Salmo salar cDNA clone ssal\_evd\_523\_136\_rev 5', mRNA sequence

3 SNPs detected

A B C D E F G H I J K L M N O  cosegregation weighted

627 . . - . - - G G . G G G - - .   1/3 22.22
847 . . . . - - - T T T - - T T T   2/3 48.89
848 . . . . - - - G G G - - G G G   2/3 48.89
